# Analysis of Welded Joint Properties on an AISI316L Stainless Steel Tube Manufactured by SLM Technology

**DOI:** 10.3390/ma13194362

**Published:** 2020-09-30

**Authors:** Petr Mohyla, Jiri Hajnys, Kristýna Sternadelová, Lucie Krejčí, Marek Pagáč, Kateřina Konečná, Pavel Krpec

**Affiliations:** 1 Department of Mechanical Technology, Faculty of Mechanical Engineering, Technical University of Ostrava, 708 00 Ostrava, Czech Republic; petr.mohyla@vsb.cz (P.M.); kristyna.sternadelova@vsb.cz (K.S.); lucie.krejci@vsb.cz (L.K.); 2 Department of Machining, Faculty of Mechanical Engineering, Technical University of Ostrava, 708 00 Ostrava, Czech Republic; jiri.hajnys@vsb.cz; 3 Department of Material Engineering, Faculty of Materials Science and Technology, Technical University of Ostrava, 708 00 Ostrava, Czech Republic; katerina.konecna@vsb.cz; 4 V-NASS, A.S., Halasova 2938/1a, 703 00 Ostrava, Czech Republic; pavel.krpec@v-nass.cz

**Keywords:** selective laser melting, welded joints, microstructure, mechanical properties

## Abstract

This work is focused on the analysis of the influence of welding on the properties and microstructure of the AISI316L stainless steel tube produced by 3D printing, specifically the SLM (Selective Laser Melting) method. Both non-destructive and destructive tests, including metallographic and fractographic analyses, were performed within the experiment. Microstructure analysis shows that the initial texture of the 3D print disappears toward the fuse boundary. It is evident that high temperature during welding has a positive effect on microstructure. Material failure occurred in the base material near the heat affected zone (HAZ). The results obtained show the fundamental influence of SLM technology in terms of material defects, on the properties of welded joints.

## 1. Introduction

The trend in recent years has become to automate the process of product production in connection with the Industry 4.0 concept. This concept includes additive technologies (AM), which replace traditional conventional technologies such as machining, casting, forming, etc. AM technologies initially included only polymeric materials [1], over time they spread to the metals sector, making them interesting for a wider range of industries. The most widespread and at the same time very complex AM method of metal production is called Selective Laser Melting (SLM). The SLM method, like other AM methods, is based on a default 3D model, which is divided into 2D layers in special slicer SW (software). These layers are then melted to each other (layer-by-layer) directly from metallic powder by a high-energy source (laser or electron beam). The layer thickness is usually in the range of 20 to 60 µm [2]. With the correct printing parameters, a very good relative density can be achieved with components produced in this way [3].

For printers using SLM methods, there are several technological barriers that prevent the production of components that are too large. For example, in a large building chamber, it is difficult to properly regulate the flow of inert gas; it is also a requirement to use a large amount of powder and, last but not least, compliance with dimensional tolerances [4]. The solution to this problem may be a suitable division of the part and subsequent joining using a welding, soldering or gluing technique. Due to the fact that most of the available printable materials in powder form by the SLM method are weldable, it offers to deal with and further develop the welding technique. 

A few studies have already dealt with this issue. Kuryntsev [5] investigated the weldability of stainless steel produced by SLM in comparison with the same cold rolled (CR) material. He concluded that steel produced by different methods is very well weldable and that heat treatment fundamentally affects the strength of SLM welded joints. Similar research was performed by Matilainen et al. [6], when they used Laser welding as a welding method and came to the conclusion that the material produced by SLM has higher melt efficiencies at lower energy inputs (80 J/mm) than CR material. Furthermore, each material creates a different weld shape formation. Other studies have examined other types of materials. Yu et al. [7] investigated the weldability of SLMed Ti-6Al-4V alloy. They found that SLMed sheets can be well joined by laser welding together with the same material up to a cavity diameter of 200 µm. Voropaev et al. [8] observed laser welding of Inconel 718 alloy, finding the inverse effect of linear energy on the microhardness of the welded joint and also that heat treatment increased the microhardness of the welded joints by 50%. In recent years, there has also been a growing interest in welding aluminum alloys made with SLM. Scherillo et al. [9] studied Friction stir welding (FSW) of SLMed AlSi10Mg alloys. The results showed that FSW refined the grains and reduced the porosity, which led to an increase in microhardness. Zhang et al. [4] focused on the comparison of Laser and TIG (Tungsten Inert Gas) welding methods for AlSi10Mg alloy, and further investigated the weldability between the printed SLM material and the cast material. From the results, it can be concluded that the biggest problem in welding is the increased porosity of SLM components, and also that in TIG welding there is a distribution of large pores at the weld boundary.

The main subject of this article is to find a suitable solution for efficient joints of printed components using SLM technology. This technology does not allow the production of large components due to the limitations of the build chamber. The aim of the article is to better understand what happens during TIG welding of two materials printed by the SLM method. At present, there are already many articles focused on the optimization of process parameters [10,11,12], microstructure [13,14,15,16] and surface roughness [17,18,19,20,21,22] of AISI (American Iron and Steel Institute) 316L material after printing by SLM. However, there are still only few studies [23,24] dealing with the weldability of this material. For this reason, this article deals with the research of TIG weldability and the subsequent study of the microstructure and mechanical properties of AISI316L stainless steel produced by the SLM method.

## 2. Materials and Methods 

### 2.1. Powder Characterisation

Gas atomized AISI316L powder supplied by Renishaw (Wotton-under-Edge, UK) was used for experimental purposes. It is a non-magnetic austenitic stainless steel with a very small carbon content. For the determination of chemical composition (see Table 1), glow discharge optical emission spectrometry (GDOES) analysis was used, which was carried out using a Spectruma Analytic optical emission spectrometer (Spectruma Analytik GmbH, Hof, Germany) [25,26]. The bulk analysis revealed the average chemical composition of the material and was carried out under conditions of excitation of 700 V and 35 mA. The powder particle mean size was 26.33 µm with an average diameter d50 of 25.09 µm. Particle size was measured by an optical method on the KYENCE VHX-5000 (KYENCE, Osaka, Japan) device. These results were acquire from our previously research [27].

### 2.2. Settings and SLM Sample Fabrication

A Renishaw AM400 3D printer (Wotton-under-Edge, UK) was used to produce circular samples in the form of tubes, which is equipped with a laser with a maximum nominal power of 400 W. During the production of the samples, the focus size was set to 70 µm, which is the technical maximum of the device, and Argon with purity 5.0 was chosen as the inert shielding gas. Prior to inertization, the chamber was compressed. The inert gas further expelled the remaining air from the chamber and the oxygen level was kept below 1000 ppm throughout the building. Thanks to this setting, there was no oxidation of the powder during building and this also ensured the correct removal of metal fumes by gas flow. The build was prepared in QuantAM software (5.0.0.135, Renishaw, Wotton-under-Edge, UK), which is developed by Renishaw. The samples were made with dimensions of the outer diameter of 20 mm with a wall thickness of 2 mm. The SLM processing parameters are tabulated in Table 2.

### 2.3. Density Measurement

The total density of the samples was determined using the Archimedes method. The principle of the measurement was to weigh the sample on a scale in air, then immerse it in water and reweigh. The difference between the weight of an object in air and in water indicates the volume of the object. By dividing the weight of the object with its volume, we get the density of the sample. A Mettler Toledo MS204SIMO1 (Mettler-Toledo International Inc., Columbus, OH, USA) scale was used to measure and determine total density. The measurement was set to 10 replicates and the mean value and standard deviation were calculated. Samples was measured by the Archimedes’ method, see Equations (1) and (2) [28]:

(1) $$\rho =\;\frac{W_{1}}{W_{2}-\;W_{3}}\cdot \rho_{w},$$

(2) $$\rho_{Re}=\frac{\rho }{\rho_{ref.}}\cdot 100$$

where *ρ* [g cm^−3^] is the density of the sample, *W*_1_ [g] is the absolute weight of the sample in air, *W*_2_ [g] is weight of the sample after removal from the water, *W*_3_ [g] is weight of the sample in water, *ρ_w_* [g cm^−3^] is a density of water, *ρ_Re_* [g cm^−3^] is a relative density of the sample and *ρ_ref_*_._ [g cm^−3^] is the density of the reference material.

**Figure 1:**
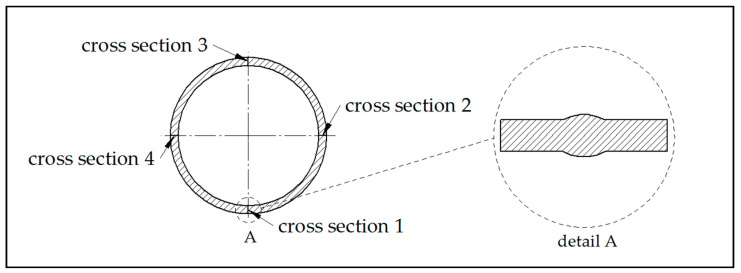
Position and orientation of cross sectioned locations for porosity measurement.

More detailed research was conducted to determine the internal porosity. The optical method with software evaluation was chosen as the evaluation method. The sample was cross-sectioned in four locations (see Figure 1), then embedded in resin and polished. The prepared sample was then scanned by an OLYMPUS GX51 microscope (Olympus, Tokyo, Japan) and the image converted to 8-bit depth for a clearer calculation of the porosity. For calculation of the total amount of pores, Image Studio 3.1 software (LI-COR Biosciences, Lincoln, NE, USA) was used for image analysis.

### 2.4. Welding of Pipes Samples

**Figure 2:**
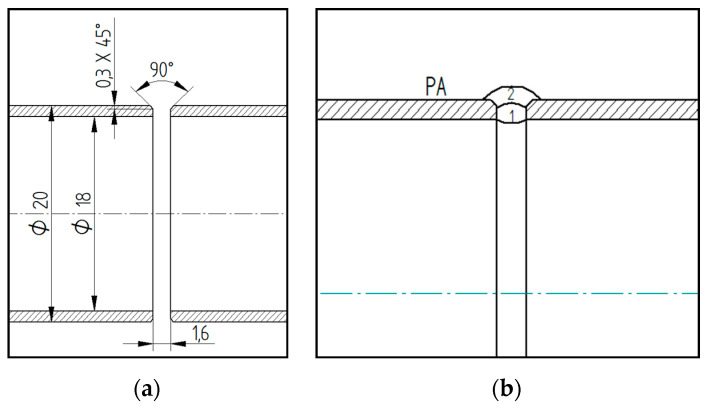
(**a**) Weld surface preparation and (**b**) method of laying weld beads.

Prior to any treatment, all samples were first heat-treated in a Clasic O816 VAK (Clasic CZ, Řevnice, Czech Republic) vacuum furnace. Annealing was applied to reduce the internal stress to 550 °C with a temperature hold of 360 min and cooling in a furnace for 12 h under vacuum [29,30]. After heat treatment, the end welds of the tubes were adapted for welding by chamfering the edges at an angle of 45° (see Figure 2) and stitching was performed followed by welding. Welding method 141 (TIG) was used for all samples, using a WLa15 electrode with a diameter of 1.6 mm. For all welded joints, a 1.6 mm diameter wire, designated 316 LSi EN 12072-19 12 3, was used. The welding position was PA (flat position with automatic tube rotation) for all tubes.

**Figure 3:**
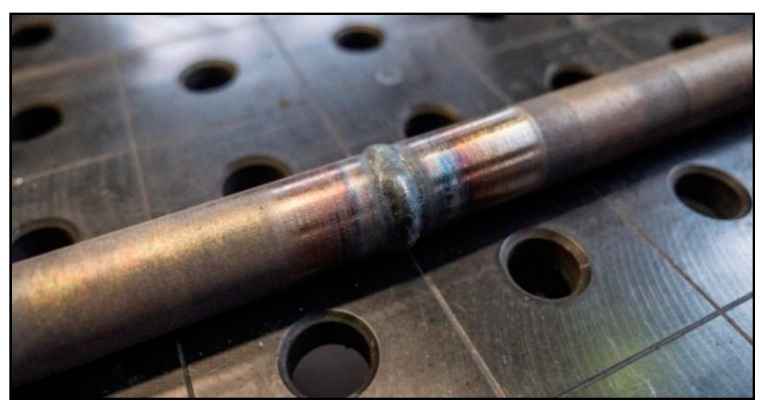
Welded joint No. 1.

Welding parameters have been set on the basis of previous experience with regard to welding thickness. The welding current for the root layer ranged from 45 to 52 A and the welding voltage ranged from 10.5 to 12.0 V. For the cover layer, the welding current was slightly increased to 48–56 A and the voltage ranged from 11.0 to 12.0 A. Argon was used as shielding gas for root protection (flow rate of 5 L/min). The welding pool was protected with the same shielding gas with a flow rate of 14 L/min. An interpass temperature of 100 °C was maintained between the root layer and the cover layer. The heat input *Q* was estimated according to Equation (3).

(3) $$Q=0.6\frac{U\cdot I}{1000\cdot v}\;,$$

where, *Q* is input heat [kJ mm^−1^], *U* is welding voltage [V], *I* is welding current [A], *v* is welding speed [mm/s]. The heat input was very low, ranging from 0.18 to 0.34 kJ mm^−1^ for the root layer and from 0.34 to 0.59 kJ mm^−1^ for the cover layer. A total of eight test welds were made, which were numbered 1 to 8. Figure 3 shows welded joint No. 1.

### 2.5. Mechanical Properties Measurement

After welding, the samples were cross-sectioned to perform microstructure and microhardness analysis. For this purpose, the samples were embedded in resin and electrolytically etched in 4% oxalic acid under conditions of 6V and 1.5A. An Olympus GX51 optical microscope (Olympus, Tokyo, Japan) with image analysis software was used for analyses. 

**Figure 4:**
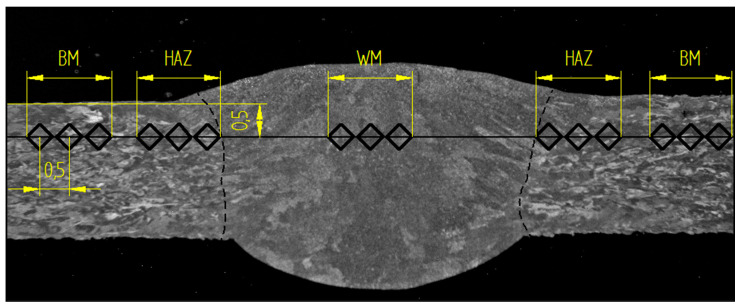
Hardness measurement methodology.

HV10 hardness was measured on a WPM Leipzig 300/436 hardness tester (WPM GmbH, Leipzig, Germany); microhardness was measured on a LECO LM 247AT microhardness tester (Leco Corporation, St Joseph, MI, USA) with a load of HV0.1. In each case, 15 punctures were made across the entire welded joint in one line. The distance between the individual indentation was set at 0.5 mm, see Figure 4. Control measurements were also made in HAZ, which were measured above and below the measurement line. 

Tensile tests were performed at +20 °C according to standard EN ISO 6892-1 [31], using the Zwick Roell Z1200H testing machine (Zwick Roell Group, Ulm, Germany). After fracture, the fracture morphology was observed by the Quanta 450 FEG (FEI Company, Hillsboro, OR, USA) scanning electron microscopy. 

## 3. Results

### 3.1. Evaluation of Base Material Porosity 

The total density was determined from the measurements and evaluation using the Archimedes method. The mean value of the printed base metal was 7.849 ± 0.01 g cm^−3^. For comparison, the total density of the homogeneous cast and rolled material of AISI316L steel is 7.999 g cm^−3^. Equation (2) is used to calculate the relative density, where the relative density is a ratio between the measured substance to the density of a given reference material [28]. The relative density in the performed experiment was 98.12%.

**Figure 5:**
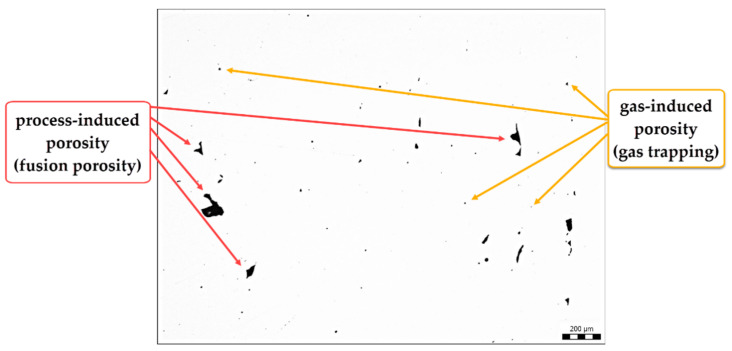
Porosity measurement with indication of the type of porosity.

Using Image analysis, the porosity of the base material was determined. The image analysis method has the disadvantage that it affects only a given 2D section and not the entire 3D volume of the sample. However, the results can be considered relevant. The average calculated porosity of the base material is 1.14%. Figure 5 shows an evaluated sample with determination of the type of porosity; more about this issue in the Discussion section, Section 4.1.

### 3.2. Evaluation of Weld Defects

**Figure 6:**
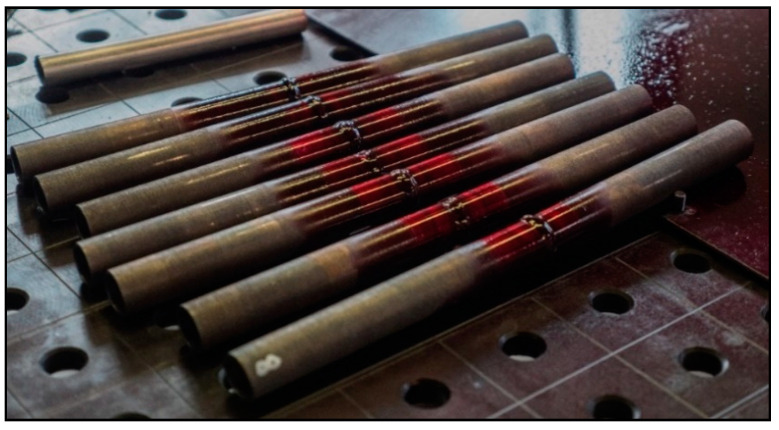
Application of detection liquid.

The capillary test was performed according to the implementation standard ČSN EN ISO 3452-1. A penetration test with the method of color indication was used, where the defects are manifested by the formation of a contrasting color indication (mostly red on a white background). Prior to testing, the samples were thoroughly cleaned from mechanical impurities and grease adhering to the surface. Subsequently, a detection liquid or penetrant bearing Pfinder 860 (Pfinder KG, Böblingen, Germany) was applied. It was a water-washable colored penetrant with a minimum exposure time of 10 min. After the exposure time, the dried liquid was wiped off so that it was not visible on the sample and remained placed only in any defects (see Figure 6). Since the sample was made using 3D printing technology, there were pores on the surface from which it was difficult to exclude the liquid.

The indication was developed by spraying the Pfinder 871 developer, which created a white layer showing defects in the weld metal or base material. For all recorded results, Table 3 shows the occurrence of defects.

**Figure 7:**
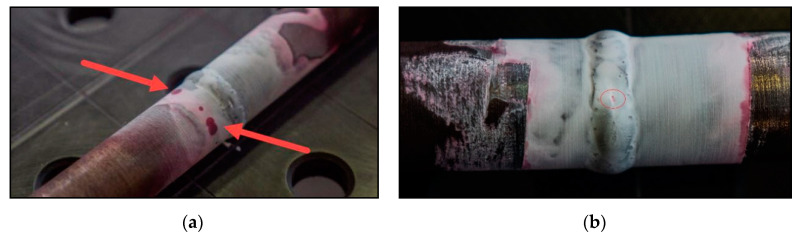
(**a**) Welded joint No. 3, defects indicated by penetrant test; (**b**) crater crack on welded joint No. 1

In sample No. 3, pores occurred around the entire circumference of the tube, which can be seen in Figure 7a. As for the characteristics of defects, we can classify them into round indications, which can be a defect of gas cavities—linear porosity (No. of Imperfections 2014 according to EN ISO 6520). This defect could have arisen during manufacture during printing, since the production is carried out in layers, i.e., from the bottom up. It was in these layers that the defect occurred due to an unknown cause.

On sample No. 1, a crater crack of small dimensions was formed (see Figure 7b), which according to the ČSN EN ISO 6520 standard is marked with the number 104. A crack is a specific type of hot crack that occurs when welding is stopped abruptly and quickly. They are formed in the crater cavity and are caused by shrinkage during the solidification of the weld metal. 

### 3.3. Evaluation of Mechanical Properties

#### 3.3.1. Metallographic Analysis

**Figure 8:**
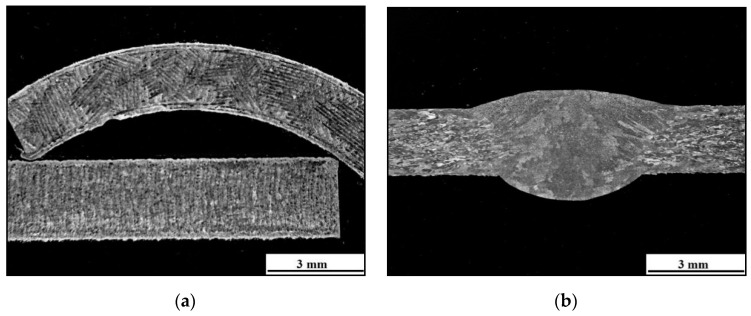
(**a**) Transverse section (on the top) and longitudinal section (on the bottom) of the base material; (**b**) macrostructure of the welded joint.

For macroscopic tests, sample No. 4 was selected. Figure 8a shows the macrostructure of the tube in the longitudinal and transverse sections. In this image, a characteristic pattern of 3D printing can be observed, which is guided by the chosen scanning strategy, which in this case is the chessboard. Figure 8b shows the macrostructure of the welded joint. 

**Figure 9:**
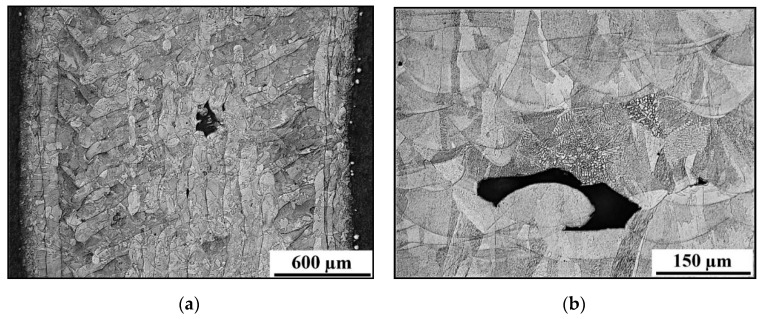
(**a**) Microstructure of the base metal; (**b**) Microstructure with process defects.

The microstructure of the base material is shown in Figure 9a. It is a typical SLM microstructure, formed from very little “weld beads” (a deposit of filler metal from a single welding pass, similar to in microwelding). The size of the individual layers is 50 µm. Different orientations of “weld beads” can be seen on the image. Figure 9b shows the detail of the base metal microstructure with defects.

**Figure 10:**
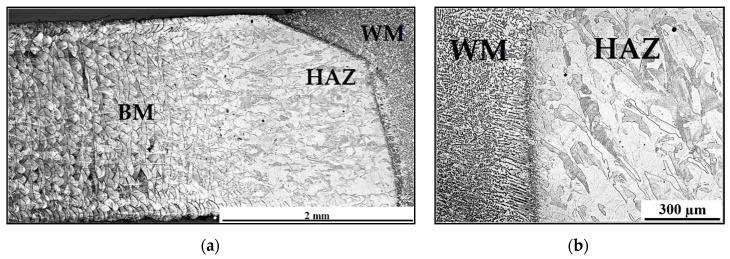
(**a**) Transition between base metal (BM) and weld metal (WM); (**b**) epitaxial growth of weld metal grains.

Figure 10a shows the transition between the base metal and the weld metal. In the heat affected zone (HAZ), the original texture of 3D printing changes to an almost homogeneous austenitic structure with a minimum of pores. The smaller the distance from the fuse boundary line, the lless the occurrence of pores in the structure is observed. The initial texture of the 3D print toward the fuse boundary disappears. The weld metal has a homogeneous austenitic microstructure with a rare occurrence of delta ferrite, otherwise with minimal defects. Figure 10b shows epitaxial growth of weld metal grains.

#### 3.3.2. Hardness and Microhardness

**Figure 11:**
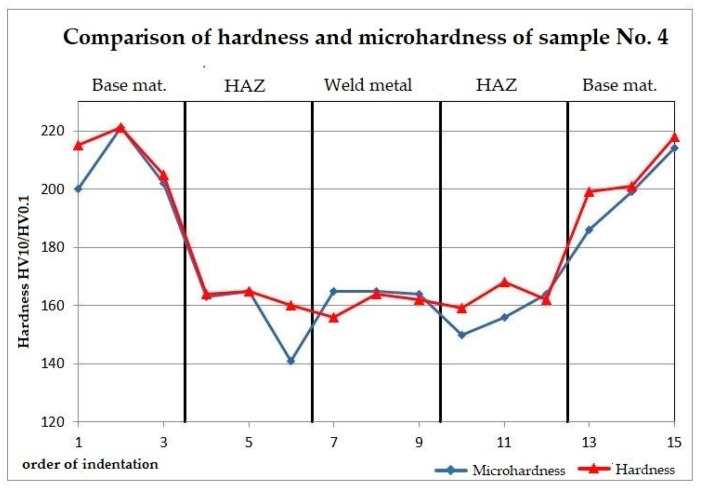
Comparison of hardness and microhardness of sample No. 4.

The trend of hardness and microhardness shows that the base material is harder than the weld metal by about 50 HV, see Figure 11. Fluctuations in the measurement in the base material could be caused by measuring at the location of the defect of the base material, such as pores. The trends of hardness and microhardness values are very similar, see Figure 11.

**Figure 12:**
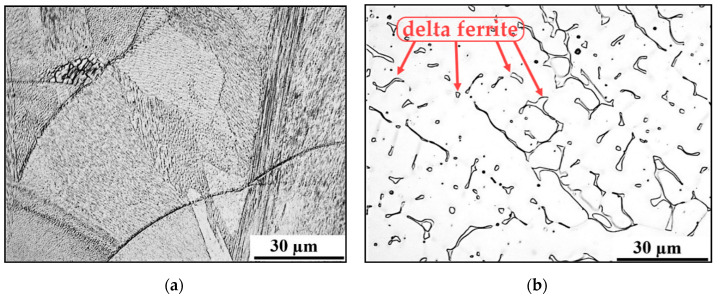
(**a**) Fine precipitates in the base material; (**b**) Austenite with delta ferrite in weld metal (all formations are delta ferrite, such as those marked with a red arrow); captured by the Olympus GX51 optical microscope, Nomarski contrast mode.

Figure 12a shows a significant dispersion of lamellar particles (precipitates) at the boundaries of austenitic grains and inside the grains. Precipitation occurred during the heat treatment of the base material. Prior to welding, annealing was performed to remove internal stress at 550 °C/360 min. In contrast, no precipitates are observed in the weld metal. The microstructure of the weld metal has dendritic morphology and is formed by austenite with delta ferrite formations, which corresponds with study [32], see Figure 12b. The HAZ microstructure is formed by austenite with a rare occurrence of globular precipitates. Most precipitates probably dissolved in this area during welding. 

#### 3.3.3. Tensile Properities

Weld joints No. 3, 5 and 6 were selected for the tensile test. All three samples were tested in the same way by the transverse tensile test; whole welded tubes were used as a sample for the test. Samples 3 and 5 experienced premature fracture. The fracture in sample No. 3 occurred before reaching the yield point outside the weld (in base metal). In sample 5, premature fracture was also indicated just above the yield point. The place of rupture was also located outside the weld. The test specimen of sample No. 6 ruptured in the heat affected zone (HAZ). This sample can therefore be considered acceptable for the research of the strength of the welded joint, the measured UTS (Ultimate tensile strength) value was 573 MPa. The obtained results are listed in Table 4.

**Figure 13:**
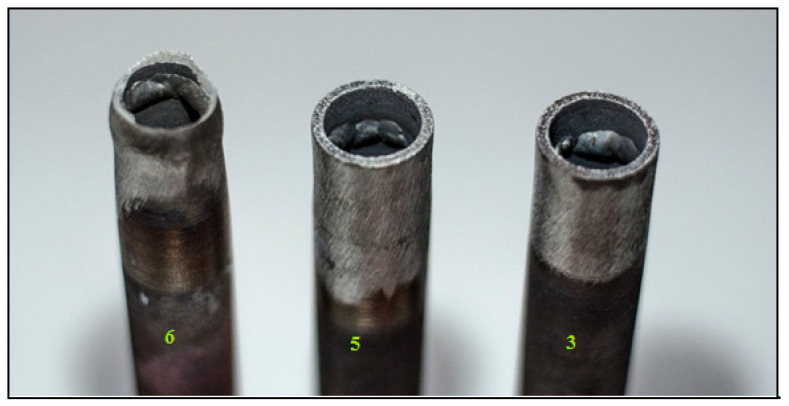
Test specimens after tensile test from the left: sample No. 6, No. 5 and No. 3.

Premature fracture of sample No. 3 was caused by a defect in the base material, which was identified by a penetrant test, see Figure 7a. The fracture occurred at the site of this defect. A similar situation appeared in sample No. 5, which also indicated premature fracture just above the yield point due to defects in the base material. These defects were partially detected by the penetrant test. Figure 13 shows broken testing specimens—samples No. 3, 5 and 6 after the tensile test; this figure also shows that the fracture point for sample No. 6 is situated in HAZ (unlike samples 3 and 5). It is apparent that only the sample 6 has reached the strength limit. 

#### 3.3.4. Fractography of Fracture Surfaces

**Figure 14:**
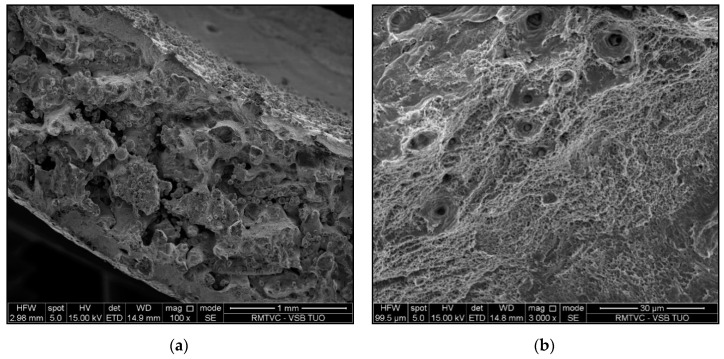

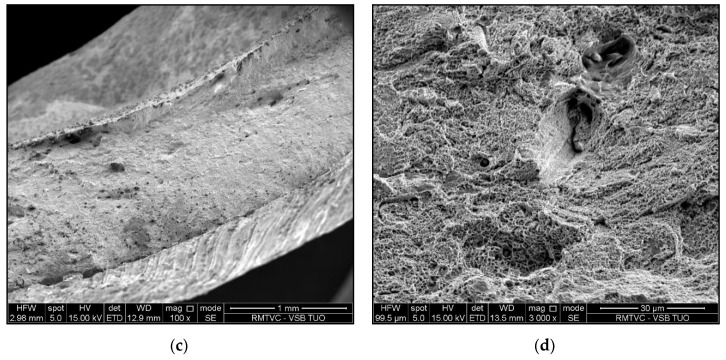
(**a**) Fracture surface of sample No. 5; (**b**) Detail of ridges on the fracture surface, sample No. 5, transcrystalline ductile fracture; (**c**) Fracture surface of sample No. 6; (**d**) Details of fracture surface in the middle of the sample, sample No. 6, transcrystalline ductile fracture and pores.

Fractography analysis was performed on samples 5 and 6 (specimens after tensile test). Scanning electron microscopy using secondary electrons (SEM-SE) was used for this analysis. Figure 14a shows fracture surface of sample 5. Most of the fracture surface is created by large, connected pores and non-molten powder particles. The ridges on the fracture surface were broken by transcrystalline ductile fracture, see Figure 14b. It should be recalled that Sample No. 5 ruptured by premature fracture. Figure 14c shows the fracture surface of sample 6, which reached the strength limit. The fracture occurred in a transcrystalline ductile manner. Seldom, larger, individual pores occur, especially in the center of the sample, see Figure 14d. 

## 4. Discussion

### 4.1. Porosity Formation

From Figure 3, it can be seen that components made using the SLM method contain internal pores. These pores are caused by insufficient or imperfect melting of the particles, or due to the entrapment of gases by surface turbulence. The porosity caused by insufficient melting (so-called fusion porosity) usually occurs along the boundaries of the layers and is characterized by its irregularity of shapes extending along the X and Y planes [33]. Pore size and occurrence are affected by process parameters (laser power, layer thickness, scanning speed and hatching distance). The cause of the porosity is insufficient scattering of the laser energy density over the entire surface of the layer, which results in non-melting of the surface of the previous layer and does not result in a coherent bond between adjacent layers. To eliminate this phenomenon, Yasa et al. [34] suggested using the re-melting method. They found that a full density of 100% could almost be achieved when each layer was scanned twice. Another possible cause of porosity is the entrapment of gas by the surface turbulent flow of molten metal in the melting bath. Deev et al. [35] states that this gas may be formed by evaporation of the material or it may be a protective atmosphere gas, but it may also be a combination thereof.

The calculated relative density using Archimedes’ law correlates with the results obtained by the Image analysis method. The weld material did not show any porosity values, so the TIG method is suitable for welding SLMed steel AISI316L.

### 4.2. Mechanical Properties Analysis

The hardness of the base material is higher compared to the weld metal and the HAZ zone also has lower hardness values. This is due to the precipitation of carbides in the base material; the subsequent TIG welding caused a partial dissolution of the carbides in the HAZ, which resulted in a reduction in hardness. 

When evaluating tensile tests, only one sample proved to be relevant (sample No. 6) when the rupture occurred in the HAZ zone, in the other samples there was a rupture outside the HAZ, which was caused by increased porosity in the production of SLM samples. The fracture surface of samples 5 and 6 differs significantly. Sample No. 5 ruptured by premature fracture. The fracture surface is created by large, connected pores and non-molten powder particles; only the ridges on the fracture surface were broken by transcrystalline ductile fracture. The fracture of sample 6 (which reached the strength limit) represents a transcrystalline ductile manner. 

## 5. Conclusions

This work is a contribution to the research and development of the joining of components produced by 3D printing, specifically produced by Selective Laser Melting (SLM), for the material AISI316L. The TIG method was used as the welding method. A total of eight welded joints were made. For individual tests (microhardness, tensile tests, porosity determination), welded samples were randomly selected on which the tests were realized. The following conclusions can be drawn from the obtained results:

1. The relative density of components manufactured by the SLM method is 98.12%.
2. The defects in the base material, which mainly include pores, have affected the mechanical properties of the welded joints so much that they initiated premature fracture before or just above the yield point.
3. Material failure occurred in the area of the base material near the heat affected zone (HAZ).
4. The microhardness is about 30% lower in the HAZ zone than in the base material.
5. In terms of microstructure, a higher temperature (closer to the fuse boundary) has a positive effect. The original texture of 3D printing changes to an almost homogeneous austenitic structure. From this point of view, heat treatment of SLM products at temperatures above 1000 °C can be recommended after 3D printing.
6. Two of the three samples broke outside the “weld zone”. This was probably because of the SLM process parameters.
7. The weld material did not show any porosity and therefore the TIG method is suitable for welding the SLMed steel AISI316L.

Further tests and analysis are required in order to confirm the experimental results and optimize part manufacturing. However, the results obtained can serve as a good scientific basis for further research in this area.

## Figures and Tables

**Table 1:** Chemical composition of AISI316L base material in Wt.%.

C	Mn	Si	P	S	Cr	Ni	Mo	W	Cu	Ti	Nb	Al	B
0.016	1.17	0.22	0.023	0.0067	17.72	14.24	2.73	0.19	0.077	0.0003	0.013	0.01	0.002

**Table 2:** Settings of process parameters for production SLM samples.

Laser Power [W]	Scanning Speed [mm/s]	Layer Thickness [µm]	Strategy
200	650	50	Chessboard

**Table 3:** Indication overview.

Sample	Indication Immediately after Spraying	Indication after 10 min
No. 1	-	Crater crack in weld metal
No. 2	-	-
No. 3	Yes	Pores in the base material around the perimeter
No. 4	-	-
No. 5	Yes	Pores in the base material around the perimeter
No. 6	-	-
No. 7	-	-
No. 8	-	Isolated defects in the base material

**Table 4:** Results of tensile test.

Sample No.	Max. Load	Tensile Strength	Place of Rupture
**-**	kN	MPa	**-**
3	33.3	267	Out of weld
5	46.1	375	Out of weld
6	70.0	573	HAZ (Heat Affected Zone)
